# Phase Ib and Expansion Study of Gemcitabine, *Nab*-Paclitaxel, and Ficlatuzumab in Patients With Metastatic Pancreatic Cancer

**DOI:** 10.1093/oncolo/oyad002

**Published:** 2023-02-18

**Authors:** Kimberly Perez, Anna M Chiarella, James M Cleary, Nora Horick, Colin Weekes, Thomas Abrams, Lawrence Blaszkowsky, Peter Enzinger, Marios Giannakis, Lipika Goyal, Jeffrey A Meyerhardt, Douglas Rubinson, Matthew B Yurgelun, Wolfram Goessling, Bruce J Giantonio, Lauren Brais, Victoria Germon, Danielle Stonely, Srivatsan Raghavan, Basil Bakir, Koushik Das, Jason R Pitarresi, Andrew J Aguirre, Michael Needle, Anil K Rustgi, Brian M Wolpin

**Affiliations:** Department of Medical Oncology, Dana-Farber Cancer Institute, Boston, MA, USA; Harvard Medical School, Boston, MA, USA; Herbert Irving Comprehensive Cancer Center, Columbia University Irving Medical Center, New York, NY, USA; Department of Medical Oncology, Dana-Farber Cancer Institute, Boston, MA, USA; Harvard Medical School, Boston, MA, USA; Biostatistics Center, Massachusetts General Hospital, Boston, MA, USA; Harvard Medical School, Boston, MA, USA; Department of Medicine, Massachusetts General Hospital, Boston, MA, USA; Department of Medical Oncology, Dana-Farber Cancer Institute, Boston, MA, USA; Harvard Medical School, Boston, MA, USA; Harvard Medical School, Boston, MA, USA; Department of Medicine, Massachusetts General Hospital, Boston, MA, USA; Department of Medical Oncology, Dana-Farber Cancer Institute, Boston, MA, USA; Harvard Medical School, Boston, MA, USA; Department of Medical Oncology, Dana-Farber Cancer Institute, Boston, MA, USA; Harvard Medical School, Boston, MA, USA; Harvard Medical School, Boston, MA, USA; Department of Medicine, Massachusetts General Hospital, Boston, MA, USA; Department of Medical Oncology, Dana-Farber Cancer Institute, Boston, MA, USA; Harvard Medical School, Boston, MA, USA; Department of Medical Oncology, Dana-Farber Cancer Institute, Boston, MA, USA; Harvard Medical School, Boston, MA, USA; Department of Medical Oncology, Dana-Farber Cancer Institute, Boston, MA, USA; Harvard Medical School, Boston, MA, USA; Department of Medicine, Massachusetts General Hospital, Boston, MA, USA; Department of Medicine, Massachusetts General Hospital, Boston, MA, USA; Department of Medical Oncology, Dana-Farber Cancer Institute, Boston, MA, USA; Department of Medical Oncology, Dana-Farber Cancer Institute, Boston, MA, USA; Department of Medical Oncology, Dana-Farber Cancer Institute, Boston, MA, USA; Department of Medical Oncology, Dana-Farber Cancer Institute, Boston, MA, USA; Harvard Medical School, Boston, MA, USA; Herbert Irving Comprehensive Cancer Center, Columbia University Irving Medical Center, New York, NY, USA; Division of Gastroenterology, Washington University School of Medicine, St. Louis, MO, USA; Perelman School of Medicine, University of Pennsylvania, Philadelphia, PA, USA; Department of Medical Oncology, Dana-Farber Cancer Institute, Boston, MA, USA; Harvard Medical School, Boston, MA, USA; AVEO Oncology, Cambridge, MA, USA; Herbert Irving Comprehensive Cancer Center, Columbia University Irving Medical Center, New York, NY, USA; Department of Medical Oncology, Dana-Farber Cancer Institute, Boston, MA, USA; Harvard Medical School, Boston, MA, USA

**Keywords:** phase Ib clinical trial, gemcitabine, *nab*-paclitaxel, ficlatuzumab, metastatic pancreatic cancer

## Abstract

**Background:**

In preclinical pancreatic ductal adenocarcinoma (PDAC) models, inhibition of hepatocyte growth factor (HGF) signaling using ficlatuzumab, a recombinant humanized anti-HGF antibody, and gemcitabine reduced tumor burden.

**Methods:**

Patients with previously untreated metastatic PDAC enrolled in a phase Ib dose escalation study with 3 + 3 design of 2 dose cohorts of ficlatuzumab 10 and 20 mg/kg administered intravenously every other week with gemcitabine 1000 mg/m^2^ and albumin-bound paclitaxel 125 mg/m^2^ given 3 weeks on and 1 week off. This was followed by an expansion phase at the maximally tolerated dose of the combination.

**Results:**

Twenty-six patients (sex, 12 male:14 female; median age, 68 years [range, 49-83 years]) were enrolled, 22 patients were evaluable. No dose–limiting toxicities were identified (*N* = 7 pts) and ficlatuzumab at 20 mg/kg was chosen as the maximum tolerated dose. Among the 21 patients treated at the MTD, best response by RECISTv1.1: 6 (29%) partial response, 12 (57%) stable disease, 1 (5%) progressive disease, and 2 (9%) not evaluable. Median progression-free survival and overall survival times were 11.0 months (95% CI, 7.6-11.4 months) and 16.2 months (95% CI, 9.1 months to not reached), respectively. Toxicities attributed to ficlatuzumab included hypoalbuminemia (grade 3, 16%; any grade, 52%) and edema (grade 3, 8%; any grade, 48%). Immunohistochemistry for c-Met pathway activation demonstrated higher tumor cell p-Met levels in patients who experienced response to therapy.

**Conclusion:**

In this phase Ib trial, ficlatuzumab, gemcitabine, and albumin-bound paclitaxel were associated with durable treatment responses and increased rates of hypoalbuminemia and edema.

## Teaser

This article evaluates the safety profile and clinical efficacy of ficlatuzumab (AV-299), in combination with standard systemic chemotherapy of gemcitabine and albumin-bound paclitaxel, in patients with metastatic pancreatic ductal adenocarcinoma.

**Implications for Practice**

Dysregulation of the hepatocyte growth factor (HGF)/c-Met signaling pathway can lead to aberrant cell proliferation, drug resistance, and promotion of cell migration and invasion. In pre-clinical pancreatic adenocarcinoma models, ficlatuzumab, an anti-HGF monoclonal antibody reduced tumor burden in combination with gemcitabine. In this multi-center, phase Ib dose escalation study, 2 dose cohorts of ficlatuzumab (10 and 20 mg/kg intravenously every 2 weeks) were administered with gemcitabine and *nab*-paclitaxel in patients with treatment naïve, metastatic pancreatic adenocarcinoma. The combination of ficlatuzumab with gemcitabine and *nab*-paclitaxel was safe and demonstrated longer survival times when compared to historical controls. The most common adverse events attributable to ficlatuzumab were hypoalbuminemia and peripheral edema. Patients who experienced a partial response to treatment had higher pre-treatment tumor p-Met levels. Further evaluation is warranted of therapies targeting the HGF/c-Met signaling pathway in pancreatic cancer.

## Background

Pancreatic ductal adenocarcinoma (PDAC) is a highly aggressive disease with poor clinical outcomes. In a recent report, Li et al identified c-Met as a potential novel human pancreatic cancer stem cell marker.^1^ Notably, c-Met is overexpressed in pancreatic cancer and appears to play an integral role in tumor growth and metastatic potential.^1-4^ In orthotopic patient-derived xenograft (PDX) models, treatment with cabozantinib (c-Met inhibitor) has been associated with a reduction in tumor size, as well as reduced metastasis formation.^1^ Recently, published work provided further evidence supporting the role of c-Met, and its ligand hepatocyte growth factor (HGF), in PDAC tumorigenesis and identified HGF as a novel transcriptional target of Prxx1b.^5^ Importantly, HGF inhibition in combination with gemcitabine reduced primary tumor volume and eliminated metastatic disease in preclinical pancreatic adenocarcinoma models.^5-7^

Ficlatuzumab, a humanized immunoglobulin G1 monoclonal antibody that targets HGF, is a HGF-c-Met inhibitor which has been evaluated in multiple studies in patients with advanced solid tumors.^8-11^ These studies confirmed a modest side effect profile and a treatment dose of 20 mg/kg once every 2 weeks of a 14-day cycle. The objective of the current study was to evaluate the safety profile and clinical efficacy of ficlatuzumab (AV-299), in combination with standard systemic chemotherapy of gemcitabine and albumin-bound paclitaxel in patients with metastatic PDAC.

## Methods

### Patients

Patients were eligible if they were 18 years of age or older and had histologically or cytologically confirmed, measurable by RECIST version 1.1, previously untreated metastatic PDAC. Other inclusion criteria were an Eastern Cooperative Oncology Group (ECOG) performance status score of 0 or 1 and adequate bone marrow (granulocyte count >/= 1500 per cubic millimeter; and platelet count >/= 100 000 per cubic millimeter), liver (bilirubin </= 1.5 times the upper limit of the normal range), and renal (creatinine >/= 1.5 times the upper limit of the normal range OR creatinine clearance >/= 60 mL/minutes/1.73 m^2^ for participants with creatinine levels above 1.5× upper limit of normal) function.

Prior systemic treatments were permitted if they received (1) adjuvant treatment after surgical resection with single-agent gemcitabine, gemcitabine/capecitabine, or 5-fluorouracil/leucovorin if completed >12 months before enrollment; (2) adjuvant radiation with or without chemotherapy sensitization with 5-fluorouracil, capecitabine, or gemcitabine if completed > 12 months before enrollment; (3) neoadjuvant treatment prior to surgical resection with single-agent gemcitabine or 5-fluorouracil/leucovorin/oxaliplatin/irinotecan if completed >12 months before enrollment.

Exclusion criteria were an endocrine or acinar cell pancreatic carcinoma, previous chemotherapy for locally advanced or metastatic pancreatic cancer, radiotherapy for measurable lesions, concurrent use of any other anti-cancer therapy, cerebral metastases, history of another major cancer, active infection, chronic diarrhea, pre-existing peripheral neuropathy (CTCAE grade 2 or higher), clinically significant history of cardiac disease, and pregnancy or breast-feeding.

### Treatment Program

**Figure 1.**
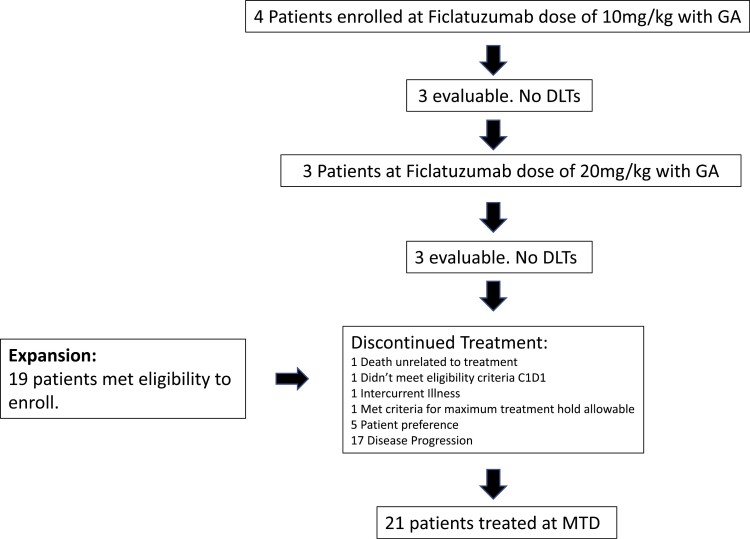
Diagram of patients enrolled.

This was a multi-center, phase Ib dose escalation study with a 3 + 3 design conducted at 2 Harvard Cancer Center sites, namely Dana-Farber Cancer Institute/Brigham Cancer Center and Massachusetts General Hospital (Fig. 1). There were 2 dose cohorts of ficlatuzumab (AV-299) administered on days 1 and 15, with gemcitabine (1000 mg/m^2^) and albumin-bound (*nab*-) paclitaxel (125 mg/m^2^) given 3 weeks on and 1 week off, followed by an 18-patient expansion phase at maximally tolerated dose (MTD) for safety evaluation. The dose cohorts of ficlatuzumab included dose level 1 at 10 mg/kg and dose level 2 at 20 mg/kg. The 20 mg/kg dose was taken forward to the expansion phase. In the dose escalation portion, a cohort of 3 patients received dose level 1 of ficlatuzumab on days 1 and 15. Progression to the next dose level was determined by the number of patients who experienced a dose limiting toxicity (DLT) event. If 0 of 3 patients experienced a DLT, then the next 3 patients enrolled received dose level 2. If 1 of 3 of the patients experienced a DLT, then the next 3 patients enrolled received the same dose level 1. If 2 or more patients experienced a DLT, then the next 3 patients enrolled received dose level −1. Dose modifications were based on clinical descriptions and grading scales found in the revised NCI Common Terminology Criteria for Adverse Events (CTCAE) version 4.0.

Radiologic tumor assessments included contrast-enhanced CT scan of the chest, abdomen and pelvis or MRI abdomen, and pelvis with contrast-enhanced CT chest. Radiologic response was classified according to RECISTv1.1. Tumor markers, carcinoembryonic antigen (CEA) and CA 19-9, were followed every 4 weeks, or at the initiation of each chemotherapy cycle. Patients were followed for 6 months after removal from protocol therapy or until death, whichever occurred first. Participants removed from protocol therapy for unacceptable adverse event(s) were followed until resolution or stabilization of the adverse event.

Blood samples were taken to assess circulating HGF measurements on day 1 of cycles 1 and 2. The Viracor Eurofins Clinical diagnostics plasma assay for quantification of HGF is a sandwich ELISA performed in a microtiter plate format. A standard curve is used to calculate the concentration of HGF in each test sample. The assay range is 0.6-24 ng/mL.^12^

Pre-treatment tissue biopsies, which consisted of 2 needle cores for formalin-fixation, paraffin embedding, were collected to analyze pre-treatment histology and c-Met pathway activation. Initial analysis by immunohistochemistry (IHC) of HGF, c-MET, and p-MET was performed to assess presence of direct targets of ficlatuzumab.^11,13,14^ A follow-up IHC analysis was performed to assess impact of HGF and MET inhibition on downstream c-MET pathway activation, which included analysis of p-ERK, p-Akt, and P-S6k.^8^*H*-scores were calculated for IHC stains using the formula: (%weak-stained cells) × 1 + (% intermediate-stained cells) × 2 + (% strong-stained cells) × 3, with a potential range of 0-300.

### Statistical Analysis

This study evaluated the safety and tolerability of ficlatuzumab when given with standard chemotherapy with the aim of determining the maximum tolerated dose (MTD) of ficlatuzumab in combination with gemcitabine and *nab*-paclitaxel. A standard 3 + 3 design with cohorts of 3-6 evaluable patients at 2 dose levels was used to determine the MTD. An additional 18 evaluable patients were enrolled at the MTD to further explore safety, tolerability, clinical outcomes, and pharmacokinetics. Toxicities were classified and graded using CTCAE version 4.0, and treatment-related toxicity rates are reported by type and grade among all patients who received any treatment while enrolled. Clinical outcomes including response rate (RR, per RECISTv1.1), progression-free survival (PFS), and overall survival (OS) were evaluated as secondary outcomes among patients treated at the MTD. PFS was defined as time from enrollment to disease progression, death, or last follow-up, and PFS and OS were analyzed using the Kaplan-Meier method. The association between toxicities and clinical outcomes was assessed via Fisher’s exact test for RR and Cox proportional hazards models for PFS and OS. Exploratory analyses examined the relationship between clinical outcomes and pre-treatment IHC staining H-scores using Wilcoxon rank sum test for RR and Cox models for PFS and OS. A significance level of 5% was used for all analyses without correction for multiple comparisons. Statistical analyses were performed using SAS v9.4.

The study (NCT03316599) was approved by the Dana-Farber Cancer Institute institutional review board and conducted in accordance with the Declaration of Helsinki. All patients provided written informed consent before any study procedures.

## Results

### Patient Characteristics

Between January 2018 and April 2019, a total of 26 patients enrolled in the study. Baseline characteristics of the patients are listed in Table 1. The median age of the patient population was 68 years, and 46% were male. All patients had an ECOG performance status of 0 or 1. The most common sites of metastatic disease included liver (81%), lymph nodes (31%), and lung (15%).

**Table 1.** Demographics.

Number of patients	26
Age, median (range)	68 (49-83)
Sex, *n* (%)	
Male	12 (46)
Female	14 (54)
Race, *n* (%)	
White	24 (92)
Black or African American	1 (4)
Other	1 (4)
Ethnicity, *n* (%)	
Hispanic or Latino	1 (4)
Non-Hispanic	25 (96)
ECOG, *n* (%)	
0	10 (38)
1	16 (61)
CA 19-9, *n* (%)	
<35 U/mL	4 (12)
>35 U/mL	22 (88)
Number of metastatic sites, median (range)	1 (1-3)
Sites of disease, *n* (%)	
Liver	21 (81)
Lung	4 (15)
Lymph nodes	8 (31)
Stomach	2 (8)
Gastrointestinal tract	1 (4)
Spleen	1 (4)
Intra-abdominal	1 (4)
Other^1^	3 (12)

^1^Other metastatic sites: left renal artery, peritoneum, omentum.

### Treatment, DLT, and Determination of Maximum Tolerated Dose (MTD)

In the phase Ib study, a minimum of 3 enrolled patients per dose level was planned. No DLTs were reported in the first 3 patients enrolled at dose level 1 (ficlatuzumab 10 mg/kg) or 3 subsequent patients enrolled at dose level 2 (ficlatuzumab 20 mg/kg). Therefore, the regimen of gemcitabine 1000 mg/m^2^, albumin-bound paclitaxel 125 mg/m^2^ combined with ficlatuzumab 20 mg/kg was identified as the maximum tolerated dose and this regimen was used for the expansion study.

In total, 25 patients were assessable for toxicity, as summarized in Table 2; 1 patient did not meet eligibility criteria on cycle 1 day 1, and did not receive treatment. There were no grade 4 or 5 toxicities attributable to ficlatuzumab. The most common grades 2 and 3 toxicities attributable to ficlatuzumab were edema (48%) and hypoalbuminemia (52%). Among the 19 with any grade edema 4 (23%) had a dose reduction in ficlatuzumab that was concurrent with or after the earliest report of edema. Among the 17 with any grade hypoalbuminemia, 4 (24%) had a dose reduction in ficlatuzumab that was concurrent with or subsequent to the earliest report of hypoalbuminemia.

**Table 2.** Adverse toxicity profile (CTCAE v4.0) attributed to ficlatuzumab (*N* = 25).

Toxicity	Grade 2 *N* (%)	Grade 3 *N* (%)
Alanine aminotransferase increased		1 (4)
Anemia		2 (8)
Anorexia	2 (8)	
Aspartate aminotransferase increased	1 (4)	1 (4)
Dehydration		1 (4)
Diarrhea	1 (4)	
Dizziness	1 (4)	
Edema limbs	10 (40)	2 (8)
Fatigue	5 (20)	1 (4)
Fever	1 (4)	
Generalized muscle weakness	2 (8)	
Hypoalbuminemia	9 (36)	4 (16)
Hypomagnesemia		1 (4)
Hypophosphatemia	1 (4)	
Lymphedema	1 (4)	
Malaise	1 (4)	
Nausea	1 (4)	1 (4)
Neutrophil count decreased	1 (4)	
Pruritus	1 (4)	
Platelet count decreased		1 (4)
Sepsis		1 (4)
Sore throat	1 (4)	
Urinary tract infection		1 (4)
Vomiting	1 (4)	

### Secondary Endpoints: Response Rate, Progression-Free Survival, Overall Survival

**Figure 2.**
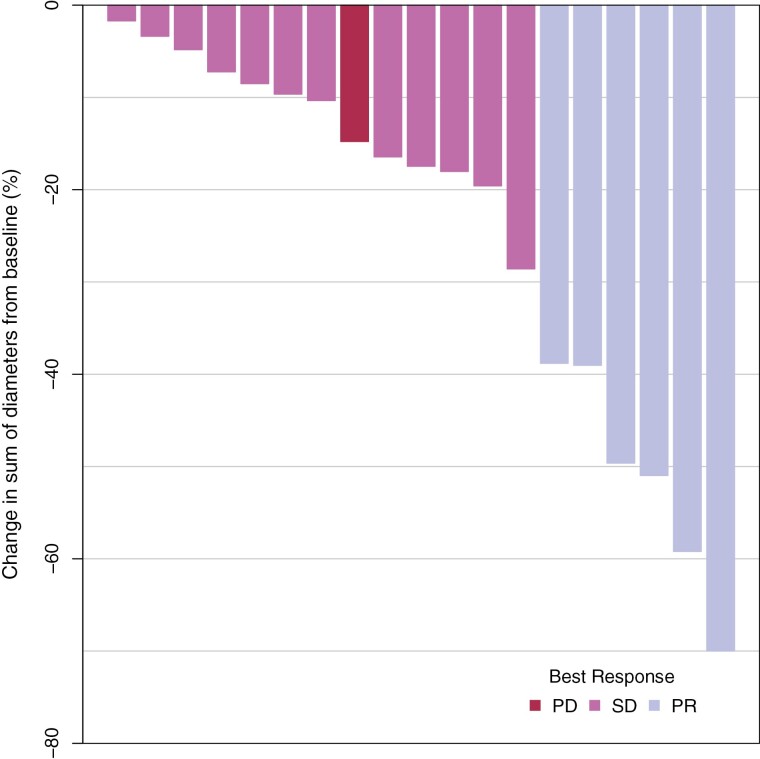
Best response by RECIST 1.1. *The one patient noted as a PD had a target lesion that met criteria for stable disease, but a non-target lesion fulfilled criteria for PD, so the overall response was PD.

**Figure 3. (:**
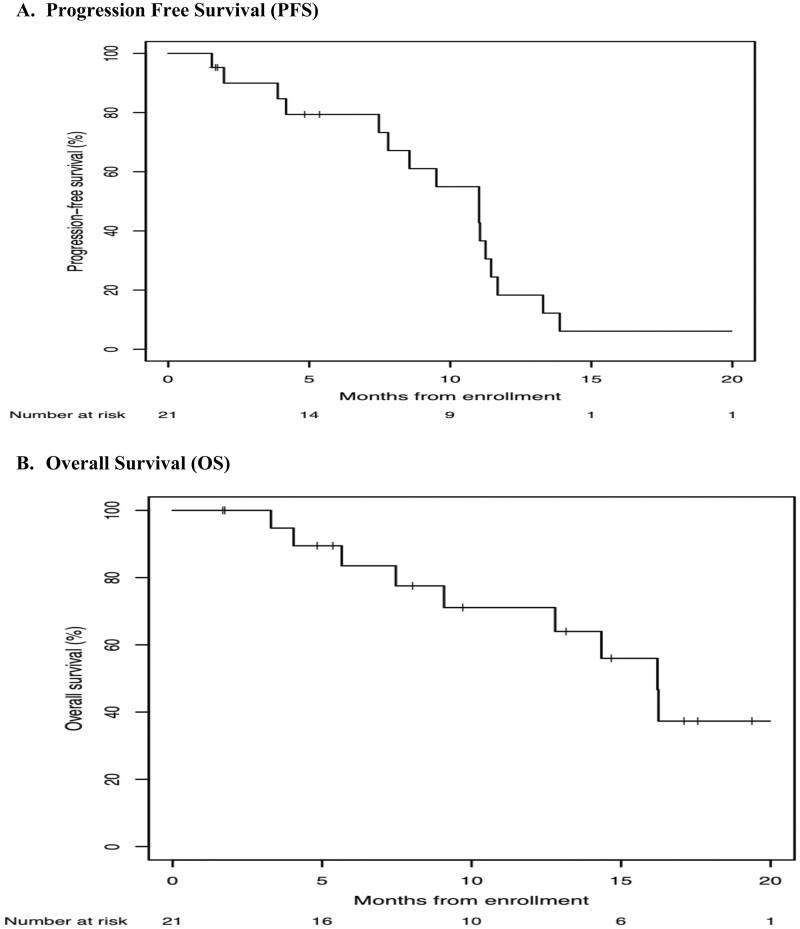
**a,b**) Kaplan–Meier survival distribution in metastatic pancreatic adenocarcinoma patients: progression-free survival (PFS) and overall survival (OS) of evaluable cohort.

Of the 21 patients treated at the MTD, 19 patients met criteria for response assessment per protocol. Two patients were not evaluable because they withdrew from therapy or discontinued treatment due to physician decision. Best responses by RECIST criteria were: 6 (29%) partial responses; 12 patients (57%) with stable disease, and 1 (5%) patient with progression prior to completion of 1 cycle of therapy (Fig. 2). Among 21 patients treated at the ficlatuzumab 20 mg/kg dose, the median PFS was 11.0 months (95% CI, 7.5-11.4 months), and median OS was 16.2 months (95% CI, 9.1 to not reached in months) (Fig. 3).

### Treatment Exposure

The median duration of treatment was 7.1 months (range, 0-24.9 months); and median number of cycles started was 7 cycles (range, 1-27). Patients were followed for 6 months after removal from protocol therapy or until death, whichever occurred first. The median follow-up time was 9.1 months (range, 0.3-26.3 months). Among the 25 patients, 48% of patients required reductions in *nab*-paclitaxel dose, 52% in gemcitabine dose, and 20% in ficlatuzumab dose over the course of treatment. In total, 30% of all doses of the combination of 3 drugs administered during the study were at the full dose for all 3 drugs. Reasons for coming off treatment included death (*n* = 1, 4%), disease progression (*n* = 16, 64), intercurrent illness (*n* = 1, 4%), withdrawal from therapy (*n* = 5, 20%), physician decision (*n* = 1, 4%), and maximum treatment hold time allowable surpassed (*n* = 1, 4%).

### Association Between Toxicities and Outcomes

Since 52% of participants developed any grade hypoalbuminemia and 48% developed any grade peripheral edema while on therapy, an analysis was performed to assess the association between these toxicities and clinical outcomes. Across RR, PFS, and OS, no statistically significant associations were identified among patients who experienced hypoalbuminemia or peripheral edema, although the 95% CIs were wide (Table 3).

**Table 3.** Association between toxicity and RR, PFS, and OS.

Toxicity	Response rate^1^		Progression-free survival^2^		Overall survival^2^	
	% w/PR	*P*-value	HR (95% CI)	*P*-value	HR (95% CI)	*P*-value
Hypoalbuminemia						
Present (*n* =1 7)	33% (5/15)	>.999	0.98 (0.35, 2.77)	.971	1.65 (0.38, 7.12)	.500
Absent (*n* = 8)	29% (2/7)		Ref		ref	
Edema						
Present (*n* = 19)	33% (6/18)	>.999	2.05 (0.45, 9.46)	.357	2.64 (0.34, 20.8)	.356
Absent (*n* = 6)	25% (1/4)		Ref		ref	

^1^Three patients who were not evaluable for response are excluded.

^2^For PFS/OS analyses, hypoalbuminemia and edema were treated as time-dependent covariates.

Abbreviations: HR, hazard ratio; PR, partial response.

### Correlative Analysis

We evaluated 11 serum sample pairs for HGF prior to initiation of treatment on days 1 and 1 of cycle 2. Of the 11 pairs, 9 were evaluable for response at MTD. There was no association between C1D1 HGF values and best response (*n* = 8 since 1 patient was not evaluable for response). The median HGF level was 0.4 (IQR: 0.4-2.5) among the patients that met RECIST criteria for PR and 0.4 (IQR: 0.4-0.6) among the patients with stable or progressive disease (*P* = 1.0000). Nor was there an association between C1D1 HGF values and PFS (HR = 1.28, 95% CI, 0.65-2.52, *P* = .4840) or OS (HR = 1.47, 95% CI, 0.65-3.31, *P* = .3536). There was no association between the difference between C2D1 and C1D1 HGF values (positive difference corresponds to increase from cycles 1 to 2) and response; median (IQR) among PR: 4.0 (3.7-4.8) and median (IQR) among SD/PD: 4.8 (3.7-5.1), *P* = .7742. Nor was there an association between change in HGF from C1D1 to C2D1 and PFS (HR = 1.39, 95% CI, 0.82-2.35, *P* = .4840) or OS (HR = 1.28, 95% CI, 0.90-1.83, *P* = .1761).

**Figure 4.**
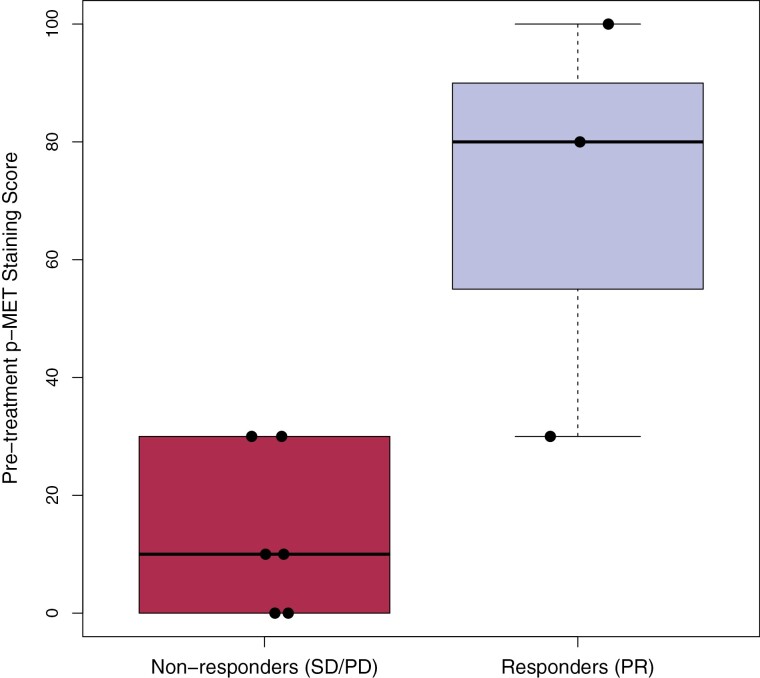
The correlation between p-Met and clinical response.

Fourteen of the patients had sufficient samples from pre-treatment tissue biopsies for immunohistochemistry analysis to characterize pre-treatment histology and assess c-Met pathway activation. Of the 14 patients, 3 received ficlatuzumab at 10 mg/kg and 11 received ficlatuzumab at 20 mg/kg. Interrogation for markers of c-Met pathway activation included analysis of HGF, c-Met, p-Met, p-ERK, p-Akt, and p-S6K expression by immunohistochemistry (IHC) in tumor cells. Among 9 patients with available response data, H-score for p-Met was associated with disease response rate (*P* = .047), with responders having a higher pre-treatment p-Met H-score (median: 80, IQR 30-100) than non-responders (median: 10, IQE: 0-30). The other measured H-scores were not significantly associated with disease response (all *P* > .05) (Fig. 4).

## Discussion

Dysregulation of the HGF/c-Met signaling pathway can lead to aberrant cell proliferation, drug resistance, and promotion of cell migration and invasion.^15-18^ In this phase Ib and expansion study, we defined a tolerable dose of ficlatuzumab when administered with gemcitabine and *nab*-paclitaxel, although the combination was associated with substantial rates of peripheral edema and hypoalbuminemia. In efficacy analyses, the addition of ficlatuzumab to gemcitabine and *nab*-paclitaxel resulted in a higher response rate and longer median survival times compared to historical controls.^19^ Notably, patients who experienced a partial response appeared to have higher pre-treatment levels of p-Met, which may mark tumors with greater HGF/c-MET pathway activation.

Edema and hypoalbuminemia are known side effects of HGF/c-Met inhibitors, including ficlatuzumab.^20-22^ In a prior monotherapy study of ficlatuzumab, serum albumin levels decreased in all patients to below the lower limit of normal by study completion. Three (16%) patients experienced serum albumin decreases to grade 3 by the end of cycle 2, and the overall incidence of any grade peripheral edema was 32%.^8^ Onartuzumab is a recombinant humanized monovalent monoclonal antibody directed against the extracellular domain of c-Met which blocks HGF binding and subsequent receptor activation.^23-26^ It is under evaluation for the treatment of a variety of solid tumors alone and in combination with chemotherapy. In a meta-analysis of 6 phase II studies and 1 phase III study conducted in patients with a variety of solid tumors, edema and hypoalbuminemia were noted as common side effects. The incidence of grade 1 to 2 peripheral edema ranged from 25% to 66%; and the incidence of grade 3 edema was reported as 14%.^27^ Any grade hypoalbuminemia ranged from 78% to 98%, with grade 3 hypoalbuminemia at 21%. There was no clear relationship between the incidence of low albumin and peripheral edema, as the incidence of low albumin was similar independent of the presence or absence of edema. The etiology of the edema was attributed to the role HGF/c-Met signaling plays in promotion of wound healing and maintenance of epithelial integrity.^28^ In studies of gemcitabine and *nab*-paclitaxel, edema has also been a reported side effect. The incidence of edema with gemcitabine has been estimated to be as high as 20%^29^; and upwards of 46% in combination with *nab*-paclitaxel.^19^ The etiology of the edema from gemcitabine and *nab*-paclitaxel is unclear, but given the higher incidence of any grade peripheral edema in our patients of 48%, we anticipate that the addition of ficlatuzumab contributed to this toxicity in the current study. Hypoalbuminemia has not been described with the combination of gemcitabine and *nab*-paclitaxel alone, and therefore it can be surmised that this toxicity is attributed to poor nutritional status and/or primarily to ficlatuzumab.

With respect to the secondary objectives, this study showed that the addition of ficlatuzumab to the combination of gemcitabine and *nab*-paclitaxel led to longer progression-free and overall survival when compared to historical controls. In the large, randomized, phase 3 study that demonstrated superiority of *nab*-paclitaxel plus gemcitabine to gemcitabine alone, the median OS was 8.5 months (95% CI, 7.89-9.53) and median PFS was 5.5 months (95% CI, 4.5-5.9) with gemcitabine and *nab*-paclitaxel.^19^ In contrast, in our cohort of patients treated at the gemcitabine and *nab*-paclitaxel plus ficlatuzumab MTD, the median OS and PFS were 16.2 months (95% CI, 9.1 months to not reached) and 11.0 months (95% CI, 7.5-11.4 months), respectively. Thus, the survival times for patients receiving the triplet combination in this study were notable, although small, early-phase studies at academic institutions may enroll patients with more favorable outcomes compared to those enrolled in large phase 3 studies.^19,30,31^

c-Met pathway activation has been associated with cancer cell proliferation and metastases,^32,33^ and activation of the c-Met pathway can be measured by tumor p-Met levels. Tabernero and colleagues previously demonstrated that ficlatuzumab resulted in a consistent decrease in tumor p-Met levels at a dose of 20 mg/kg, which confirmed that ficlatuzumab modulates HGF/c-Met pathway function in patients.^8^ Ficlatuzumab was also found to lower median levels of tumor p-ERK and p-Akt, while causing an increase in serum HGF levels.^8^ In the current study, patients who had a partial response to therapy had higher pre-treatment tumor p-Met H-scores, providing some evidence for p-Met as a potential candidate biomarker for disease response to gemcitabine and *nab*-paclitaxel plus ficlatuzumab. However, these analyses were limited by a modest sample size, and future investigation will be required to confirm the predictive ability of p-Met in patients receiving ficlatuzumab or other HGF/C-Met pathway inhibitors.

### Conclusion

In this study, the recommended dose of ficlatuzumab when combined with gemcitabine and *nab*-paclitaxel was identified as 20 mg/kg every 2 weeks. The most common side effects attributable to the addition of ficlatuzumab were hypoalbuminemia and peripheral edema, leading to dose reductions in some patients. Nevertheless, the combination of ficlatuzumab with gemcitabine and *nab*-paclitaxel demonstrated notably longer survival times when compared to historical controls. Given the potential clinical benefit for addition of ficlatuzumab to cytotoxic therapy, further evaluation of HGF/c-Met pathway inhibitors may be warranted in patients with pancreatic cancer, although alternate cytotoxic backbones that do not induce additive peripheral edema may be preferable.

## Data Availability

The data underlying this article are available in the article and in its online supplementary material.
